# Body Size-Related Thermal Acclimation and Underlying Gene Expression Mechanisms in Workers of the Red Fire Ant *Solenopsis invicta*

**DOI:** 10.3390/insects17090971

**Published:** 2026-09-20

**Authors:** Jin Xu, Rui Peng, Yu Song, Jian-Min Yang, Chao-Yang Duan, Wei Gao

**Affiliations:** 1 Yunnan Provincial Key Laboratory for Conservation and Utilization of In-Forest Resource, Southwest Forestry University, Kunming 650224, China; pengrui0517@126.com (R.P.); sy3167632311@163.com (Y.S.); 15559874689@163.com (J.-M.Y.); duanchaoyang189@163.com (C.-Y.D.); 2 Wenshan Plant Protection and Quarantine Station, Wenshan 663099, China

**Keywords:** *Solenopsis invicta*, cold acclimation, heat acclimation, olfactory reception, metabolism

## Abstract

Body size is a critical trait shaping ectotherm thermal tolerance, yet how body size mediates thermal acclimation remains poorly understood in insects. Here, we conducted 15-day daily cold (9 °C, 2.5 h) and heat (30 °C, 2.5 h) acclimation treatments on large, medium, and small *Solenopsis invicta* workers, followed by extreme temperature survival assays and comparative RNA-seq transcriptome profiling to uncover size-dependent acclimation phenotypes and molecular regulatory pathways. Survival results revealed that both cold and heat acclimation significantly boosted extreme thermal resistance of medium and small workers, whereas large workers exhibited inherently low mortality under lethal temperatures with no significant acclimation-induced survival improvement. Transcriptomic analyses showed striking body-size-specific transcriptional reprogramming: medium workers produced the largest number of differentially expressed genes (DEGs) under both acclimation regimes, followed by large workers, while small workers displayed minimal transcriptional shifts. Functional enrichment highlighted core pathways including lipid and small-molecule cryoprotectant metabolism, cuticle/membrane homeostasis, ion transport, antioxidant detoxification and sensory nervous signaling. Notably, hundreds of odorant receptor genes were sharply downregulated in acclimated medium workers, suggesting potential repression of olfactory function. Unlike acute thermal stress, long-term repeated acclimation triggered only mild transcriptional fluctuations of heat shock proteins with limited fold changes. Our findings demonstrate that thermal acclimation capacity in red fire ant workers is strongly correlated with body size, and coordinated remodeling of metabolic, osmotic, and chemosensory gene networks underpins this thermal plasticity. This body-size-dependent acclimation mechanism advances our understanding of invasive ant climate adaptation and helps predict their range expansion risks under global warming.

## Simple Summary

Body size has been recognized as an important correlate of thermal tolerance in many ectothermic insects, yet how body size mediates thermal acclimation remains unclear for invasive ant species. This study treated large, medium and small red imported fire ant (*Solenopsis invicta*) workers with 15 days of daily mild cold or heat exposure, then tested their survival under extreme temperatures and analyzed genome-wide gene expression changes. Acclimation significantly improved cold and heat resistance in medium and small workers, while large workers already had strong basal thermal tolerance with no obvious acclimation benefit. Medium workers showed the largest number of gene expression changes, involving metabolism, cuticle homeostasis, ion transport and sensory pathways. Unlike acute temperature stress, long-term acclimation caused only mild fluctuations in heat shock protein levels. Strikingly, hundreds of olfactory receptor genes were strongly downregulated in acclimated medium workers, suggesting that environmental temperature may modulate olfactory reception. These findings reveal the body-size-dependent thermal acclimation mechanism of fire ants, helping predict their invasion range expansion under global warming.

## 1. Introduction

*Solenopsis invicta* (Hymenoptera: Formicidae), the red fire ant, originates from tropical and subtropical South America [1]. This invasive ant species was first detected in Wuchuan, Guangdong Province, mainland China in 2004, and has since expanded rapidly across 12 provincial administrative regions and incurred substantial ecological and economic losses [2,3,4]. Boasting high fecundity, polyphagous feeding habits, intense interspecific competitiveness, and diverse dispersal routes [3,5,6], *S. invicta* imposes severe adverse impacts on public health, crop and forest production, indigenous biodiversity, and local ecosystems [2,4,7].

Temperature influences animal survival, development and distribution [8,9,10]. Under extreme environmental temperatures, animals undergo physiological suppression and fatal tissue damage [11,12]. Invasive insects pose severe ecological threats by disrupting biodiversity, agricultural production, and ecosystem stability [4,13]. The strong adaptability of invasive insects to fluctuating temperatures facilitates successful colonization in novel habitats [14,15]. Furthermore, climate warming is projected to expand the suitable geographic ranges of invasive insects, thereby elevating future invasion risks [16,17].

Previous studies have demonstrated that mild thermal hardening, defined as short-term (hours to days) exposure of insects to sublethal high or low temperatures, can elevate their thermal tolerance. Longer-term exposures (weeks, seasonal, or multigenerational) to sublethal temperatures are considered acclimation. Specifically, heat hardening or heat acclimation increases heat tolerance, while cold hardening or cold acclimation increases cold tolerance [18,19]. Heat or cold acclimation describes the physiological process whereby organisms progressively improve extreme-temperature tolerance via recurrent temperature exposure, constituting a core adaptive strategy enabling insects to persist under temperature stress [18,19,20]. For instance, in the common cutworm *Spodoptera litura*, prior exposure to mild thermal stress substantially enhanced adult heat resistance yet compromised reproductive performance [21]. In the fall armyworm (*Spodoptera frugiperda*), adult thermal tolerance varies with age, sex, and mating status [22], and multigenerational heat selection enhances thermal acclimation in this insect [15]. Notably, studies in a range of insect species have shown that survival under lethal temperatures increases after multiple generations of heat exposure, which indicates transgenerational heat acclimation [18,23,24,25]. However, a study in *Sitotroga cerealella* demonstrated that heat and fasting acclimation negatively affected offspring thermal tolerance; continuous dynamic heat acclimation (28–38 °C) produced stronger detrimental effects than acute static acclimation at 38 °C [19].

The molecular basis underlying insect thermal adaptation is still not fully understood, with potential regulatory pathways covering epigenetic memory, DNA methylation, and gene transcription and translation [18,23,26]. As a pivotal epigenetic modifier governing gene transcription, genome stability and cell differentiation, DNA methylation dynamically remodels transcriptional profiles upon thermal challenges in insects [18,27]. Heat exposure commonly triggers elevated transcription of heat shock proteins (Hsps), alongside their associated coding genes and transcriptional regulators [28]. Empirical evidence across multiple insect species consistently confirms heat-triggered Hsp induction: mild heat treatment elevates *Hsp90*, *Hsc90*, *Hsp70* and *Hsc70* transcripts in *S. litura* [21]; short-term thermal exposure broadly activates diverse Hsp genes in the fall armyworm [29,30], and multigenerational heat acclimation further enhanced the transcriptional response of Hsps under heat stress [15]. Thermal exposure also upregulates genes for antioxidant and detoxification enzymes and thermosensory ion channels including TRPA1 and TRPV1 [31,32]. For instance, heat treatment elevates the activities of core antioxidant enzymes (CAT, GST, SOD) in *Mythimna separata* [33], whereas it differentially modulates *TRPA1* transcription in the fall armyworm: two female-biased *TRPA1* genes are induced, yet one homolog is suppressed in males [29]. Small-molecule antifreeze protectants and proteins have been shown to play vital roles in insect cold tolerance [34]. Antifreeze small molecule protectants (SMP) including glycerol, trehalose and other sugars raise bound water content and interact with enzymes/proteins to enhance insect cold resistance [35]. Research on ants and tobacco armyworms reveals key roles of GPDH and GK in glycerol synthesis and cold tolerance mechanisms [36,37]. Low temperature induces peak levels of trehalose and trehalose synthase in wheat midges, with reduced expression of trehalose-degrading enzymes [38]. Recent studies also showed that aquaporins, a family of transmembrane channels for water and glycerol transport, are involved in insect cold and heat adaptation [39,40]. Collectively, these findings elaborate the multifaceted physiological and molecular underpinnings of insect temperature acclimation.

Thermal adaptive evolution in insects is fundamentally supported by biophysical interactions between body size and ambient temperature [11,41,42]. Nevertheless, empirical evidence demonstrates inconsistent associations between body size and thermotolerance, while the theoretical frameworks explaining such patterns continue to be debated [11,41]. Body mass shapes insect physiology and fitness: larger individuals store more water and nutrients to withstand stress, whereas big-body drawbacks involve elevated metabolism and thermal risks under opposing selective pressures [43,44,45]. Water homeostasis is critical to insect metabolism and thermoregulation under thermal stress [46,47]. Smaller insects are susceptible to desiccation under elevated temperatures owing to their high surface-area-to-volume ratios [46,47,48]. Body-size-dependent thermal tolerance, as well as physiological and molecular responses to thermal stress, have been investigated across many invertebrates [11,12,41,42,49,50,51]. Nevertheless, whether and how body size shapes insect thermal acclimation remains poorly understood.

In *S. invicta*, temperature exerts profound influences on foraging activity [52], colony development [53], brood transportation [54] and range expansion [55]. As a highly successful invader, *S. invicta* displays robust tolerance against heat and desiccation stress [46]. As noted above, *S. invicta* is native to tropical and subtropical South America, whereas most areas of China belong to the northern temperate zone; low temperature mainly restricts its expansion and colonization across China [55]. Previous studies have shown that short-term (a few hours) thermal stress decreases worker survival in *S. invicta* in a size-dependent fashion, and that size-associated disparities in transcriptional reprogramming are linked to Hsps, protective metabolites, and pathways governing water homeostasis [12,46]. As noted above, relatively few studies have investigated how body-size variation modulates thermal acclimation in insects, and this question remains incompletely understood. Building upon existing knowledge of heat-stress responses and adaptive mechanisms in insects, we hypothesized that *S. invicta* can establish thermal acclimation accompanied by size-divergent acclimation phenotypes and transcriptional reprogramming. To test this hypothesis, we performed 15-day cold and heat acclimation assays on fire ant workers of distinct body sizes. We then compared thermal tolerance traits between acclimated and control worker ants across distinct body sizes and further employed transcriptomic profiling to dissect transcriptional reprogramming under different acclimation treatments and body-size combinations.

## 2. Materials and Methods

### 2.1. Insects

*S. invicta* colonies were sampled in early July 2024 from Kunming, Yunnan Province, China (altitude, 2073 m; 25°3′33″ N, 102°45′21″ E). Complete nests containing queens were excavated to reduce stress caused by queen absence. Five medium-sized nests (designated nests 1–5) with dome-shaped mounds approximately 15 cm high and 30 cm in diameter were chosen for collection. Individual colonies were reared separately in plastic containers (25 H × 30 × 40 cm) and provided with artificial diet [56], beef sausages (Jinluo, Linyi, China), and water. Rearing conditions were set at 26 ± 1 °C, 60–80% relative humidity with a 14:10 h light:dark photoperiod. The upper 10 cm inner margin of each container was coated with talcum powder, and containers were covered by double layers of nylon mesh to prevent escape. All experiments were performed within one month following sampling. The average daily low and high temperatures in Kunming during this month reached 19 °C and 25 °C, respectively.

### 2.2. Cold and Heat Acclimation of Worker Ants of Different Sizes

*S. invicta* workers of different sizes, large (mean ± SD: 1.30 ± 0.21 mg), medium (0.60 ± 0.07 mg) and small (0.38 ± 0.03 mg), were collected from each of the 5 nests (workers from different nests were kept in separate containers for subsequent treatments) for thermal acclimation by exposing the insects to 9 °C for 2.5 h per day (cold acclimation) or 30 °C for 2.5 h per day (heat acclimation) over 15 successive days. Temperature treatments were implemented during the photophase. Ants were kept at 26 °C as described above for the rest of the experimental period. Normally reared ants maintained at 26 °C without cold or heat exposure served as controls. Food was provided to ants in all treatment groups following the aforementioned feeding protocol. All temperature treatments were carried out in artificial climate incubators (BIC-400; Shanghai Boxun, Shanghai, China) capable of regulating temperature, photoperiod (14:10 h L:D) and relative humidity (60–80%). Handheld temperature and humidity loggers were additionally used to verify actual temperature and RH conditions.

### 2.3. Survival of Acclimated Workers Exposed to Extreme Temperatures

Following a 15-day acclimation, workers of different body sizes were allowed a 24 h recovery period prior to sampling for subsequent experiments (i.e., ants were collected on day 16; control ants were collected at the same time). Each collected ant was randomly assigned to one of four temperature treatments (−1 °C, 1 °C, 35 °C, and 37 °C) for 2 h and then incubated at 26 °C for 1 h to assess mortality. All temperature treatments were performed using the above-mentioned incubators. Insects were not provided with either water or food during the stress and incubation stages. Survival assays were carried out in plastic bottles (8 cm diameter × 11 cm height). Talcum powder was applied to the upper inner rim of each bottle to prevent ant escape. Bottles were sealed with plastic lids pre-drilled with a central hole (3 cm in diameter), which was covered with two layers of nylon mesh. Active workers of distinct body sizes were randomly collected from three laboratory-reared nests and allocated to different experimental treatments. Each treatment for each body size included three biological replicates (n = 3), with each replicate originating from an independent nest (randomly selected from the five nests). Thirty ants were used per replicate.

### 2.4. Effect of Temperature Acclimation and Extreme Temperature Stress on the Transcription of Worker Ants

#### 2.4.1. Treatment and Sampling

Acclimated and control workers of different sizes were collected 24 h after the 15-day acclimation and subjected to extreme cold (1 °C) and heat (35 °C) stress for 2 h (consistent with the treatments described above), then sampled immediately after stress. In parallel, additional acclimated and control workers of distinct sizes were sampled 26 h post the 15-day acclimation without extreme temperature stress; specifically, ants remained at 26 °C for 26 h following the 15-day acclimation before sampling. Thirty ants constituted one replicate for each body size category, and three replicates derived from three independent nests were prepared per size group. Therefore, a total of 63 samples were obtained, i.e., 7 treatments (cold-acclimated ants without cold stress, cold-acclimated ants plus cold stress, heat-acclimated ants without heat stress, heat-acclimated ants plus heat stress, control ants without stress, control ants plus cold stress, and control ants plus heat stress) × 3 body sizes × 3 replicates. All collected samples were immediately frozen in liquid nitrogen and preserved at −80 °C.

#### 2.4.2. Sequencing and Assembly

Total RNA was extracted from the above experimental samples using TRIzol reagent (Invitrogen, Waltham, MA, USA). We quantified RNA concentration and purity using an Implen spectrophotometer and the Qubit RNA Assay Kit (Life Technologies, Carlsbad, CA, USA) and evaluated RNA integrity with the RNA Nano 6000 Assay Kit (Agilent, Santa Clara, CA, USA). We then constructed RNA-seq libraries with the NEBNext Ultra RNA Library Prep Kit for Illumina (New England BioLabs, Ipswich, MA, USA) and sequenced them on the Illumina HiSeq 4000 platform (Illumina Inc., Foster City, CA, USA; https://www.illumina.com, accessed on 12 November 2025), yielding 125 bp and 150 bp paired-end reads.

To obtain valid sequencing data, we used fastp (version 0.23.4) to preprocess raw reads; adapter sequences, sequences containing undetermined bases, and low-quality reads were thoroughly eliminated. Quality metrics including Q20, Q30 and GC content were computed for the final clean reads. Thereafter, the clean reads were mapped to the reference genome of *S. invicta* (UNIL_Sinv_3.0) via Hisat2 software (v2.2.1).

#### 2.4.3. Differential Expression Analysis and Functional Enrichment

We quantified gene expression using the transcripts per million (TPM) normalization method. Differential expression analysis among treatment groups was implemented via DESeq2 (v1.56.1). The *q*-value was utilized for multiple testing correction to calibrate statistical significance. Following the method of Storey [57], a gene was considered significantly differentially expressed if its *q*-value was less than 0.05 and the absolute value of log2(fold change) exceeded 1.

To explore the potential biological functions of differentially expressed genes (DEGs), we conducted Gene Ontology (GO) enrichment analysis via the GOSeq program (v2.12) and Kyoto Encyclopedia of Genes and Genomes (KEGG) pathway enrichment analysis using KOBAS software (v2.1.1). Ultimately, GO terms and KEGG pathways with *q* < 0.05 were screened and regarded as significantly enriched categories.

#### 2.4.4. Validation of RNA-Seq

Eighteen genes (12 DEGs and 6 non-DEGs) from different comparisons were selected for qPCR expression verification (Table S1). Consistent with the study’s thermal acclimation theme, the selected genes comprised Hsps, zinc-finger proteins, odorant receptor-encoding genes, and others. RNA-seq results were validated via quantitative real-time PCR (qPCR). Total RNA was isolated from samples following the aforementioned protocol, and complementary DNA (cDNA) was synthesized using the PrimeScript RT reagent Kit (Takara, Beijing, China). PCR amplifications were performed with gene-specific primers (Table S1) on a QuantStudio 7 Flex System (Thermo Fisher Scientific, Waltham, MA, USA) in a 25 μL reaction volume. The thermal cycling program consisted of an initial denaturation at 95 °C for 30 s, followed by 40 cycles of 95 °C for 5 s and 60 °C for 30 s; a melting curve analysis was subsequently carried out. Three biological replicates and three technical replicates were included for each sample. PCR amplification efficiencies were determined by generating standard curves for target and reference genes using 5-fold serial dilutions of cDNA. Melting curve profiling verified the specificity of SYBR Green amplification products. Relative expression levels of target genes were calculated using the 2^−ΔΔCT^ method [58].

### 2.5. Statistics

Two-way ANOVAs using the general linear model (GLM) were conducted to analyze the effects of acclimation (two levels) and body size (three levels) on ant mortality under stress. When significant differences were observed for both the overall model and the acclimation × size interaction, simple effects analyses (post hoc multiple comparisons) were performed using estimated marginal means with Bonferroni correction. In cases where the overall model or interaction was non-significant, additional analyses were performed using one-way ANOVAs followed by Tukey’s HSD test for >2 levels, and *t*-tests for two-group comparisons, to explore potential subtle differences across different comparison scenarios. qPCR expression data were subjected to *t*-tests. All statistical analyses were performed using SPSS 16.0 software. The significance threshold was set at α < 0.05. All data are shown as mean ± standard error (SE).

## 3. Results

### 3.1. Effect of Temperature Acclimation on Cold and Heat Tolerance of Worker Ants

**Figure 1:**
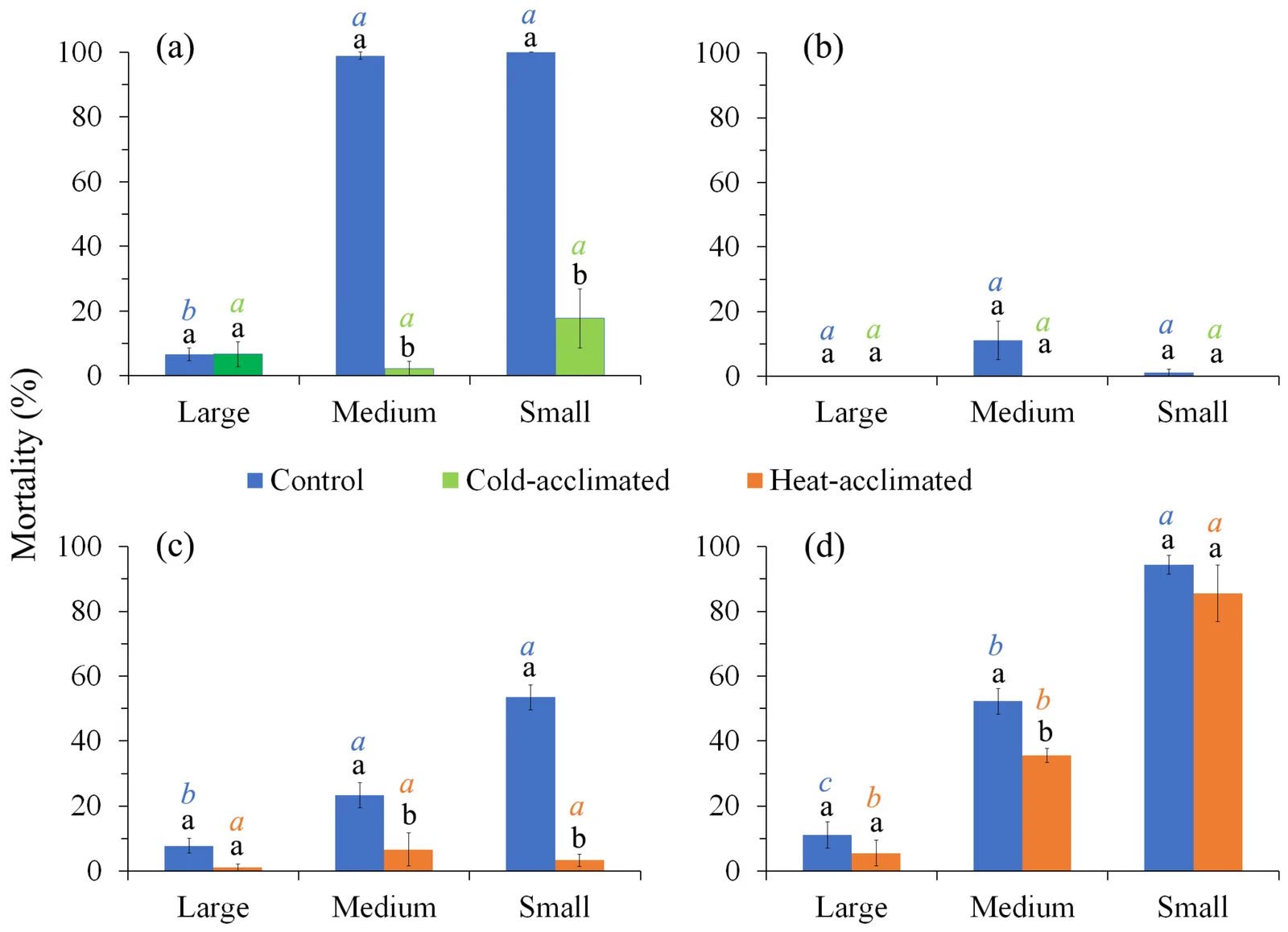
Effects of temperature acclimation on cold and heat tolerance of worker ants of different sizes. (**a**) Mortality of cold-acclimated ants stressed at −1 °C for 2 h; (**b**) Mortality of cold-acclimated ants stressed at 1 °C for 2 h; (**c**) Mortality of heat-acclimated ants stressed at 35 °C for 2 h; (**d**) mortality of heat-acclimated ants stressed at 37 °C for 2 h. Within each sub-panel (i.e., in each of the four temperature treatments), different black upright letters above bars denote significant differences (*p* < 0.05) between acclimated ants and control ants of the same body size, whereas different italic letters in the same color above bars indicate significant differences (*p* < 0.05) among worker ants of different body sizes within the same treatment conditions. Each treatment for each body size included three biological replicates (n = 3). All data are shown as mean ± standard error (SE). Detailed statistical methods and results are reported in Table S1.

Results showed that cold acclimation significantly enhanced the cold tolerance of *S. invicta* workers (Figure 1a,b). Under −1 °C, two-way ANOVA indicated significant effects (whole model, *p* < 0.0001; main effects of cold acclimation and size, *p* < 0.0001 for both; and interaction, cold acclimation × size, *p* < 0.0001) (Figure 1a; Table S2), indicating that both cold acclimation and body size significantly affected ant mortality, with a significant interaction between the two factors. Given the significant interaction, simple effects analyses were conducted for multiple comparisons, which showed that large workers have very low mortality for both acclimated and control groups, and no significant differences (*p* > 0.05) were found between them (Figure 1a); in medium and small workers, significant differences (*p* < 0.05) were found between acclimated and control groups. Under 1 °C, two-way ANOVA also indicated significant effects (whole model, *p* < 0.05; main effect of cold acclimation, *p* < 0.05), whereas the main effect of size and the cold acclimation × size interaction were non-significant (*p* > 0.05) (Figure 1b; Table S2). This result suggests that cold acclimation independently influenced the cold tolerance of workers at 1 °C. Since the interaction was non-significant, additional analyses were performed using one-way ANOVAs and *t*-tests to explore potential subtle differences across different comparison scenarios. Results showed that there were no significant differences between cold-acclimated and control groups within each body size, nor among different body sizes under the same treatment (*p* > 0.05; Figure 1b; Table S2).

Heat acclimation also significantly enhanced the heat tolerance of *S. invicta* workers (Figure 1c,d). Under 35 °C, two-way ANOVA indicated significant effects (whole model, *p* < 0.0001; main effects, *p* < 0.0001 for heat acclimation, and *p* < 0.001 for size; and *p* < 0.05 for heat acclimation × size) (Figure 1c; Table S2), indicating that both heat acclimation and body size significantly affected ant mortality, with a significant interaction between the two factors. Simple effects analyses showed that large workers have very low mortality for both acclimated and control groups, and no significant differences (*p* > 0.05) were found between them (Figure 1c); in medium and small workers, significant differences were found between acclimated and control groups. Under 37 °C, two-way ANOVA also indicated significant effects (whole model, *p* < 0.0001; main effects, *p* > 0.05 for heat acclimation, and *p* < 0.0001 for size; and *p* > 0.05 for heat acclimation × size) (Figure 1d; Table S2). This result suggests that body size independently influenced the heat tolerance of workers at 37 °C. As the interaction was non-significant, additional analyses were performed using one-way ANOVAs and *t*-tests to explore potential subtle differences across different comparisons. Significant differences between acclimated and control groups were observed in medium workers (*p* < 0.05), but not in large or small workers (*p* > 0.05); significant differences were also detected among different-sized workers under heat acclimation or control treatments (*p* < 0.05 for both; Figure 1d; Table S2).

### 3.2. RNA Sequencing and Assembly

RNA-seq yielded 39,667,076–45,453,028 clean reads from each of the 63 sequenced libraries, with Q20 and Q30 being 98.43–98.72% and 95.20–95.94%, respectively (Table S3). A total of 27,552,989–35,812,654 clean reads from each of the libraries were mapped to the genome of *S. invicta*, with the mapped ratios ranging from 68.23% to 80.78%. Principal component analysis (PCA) showed that biological replicates clustered together (Figure S1) and Pearson’s correlation coefficient showed higher correlations between biological replicates but lower correlations between treatments (Table S4), suggesting a pronounced treatment effect and data quality sufficient to support downstream differential gene expression analysis and functional enrichment analysis.

### 3.3. Cold Acclimation and Stress-Induced Transcriptional Changes and Functional Enrichment Analysis

**Figure 2:**
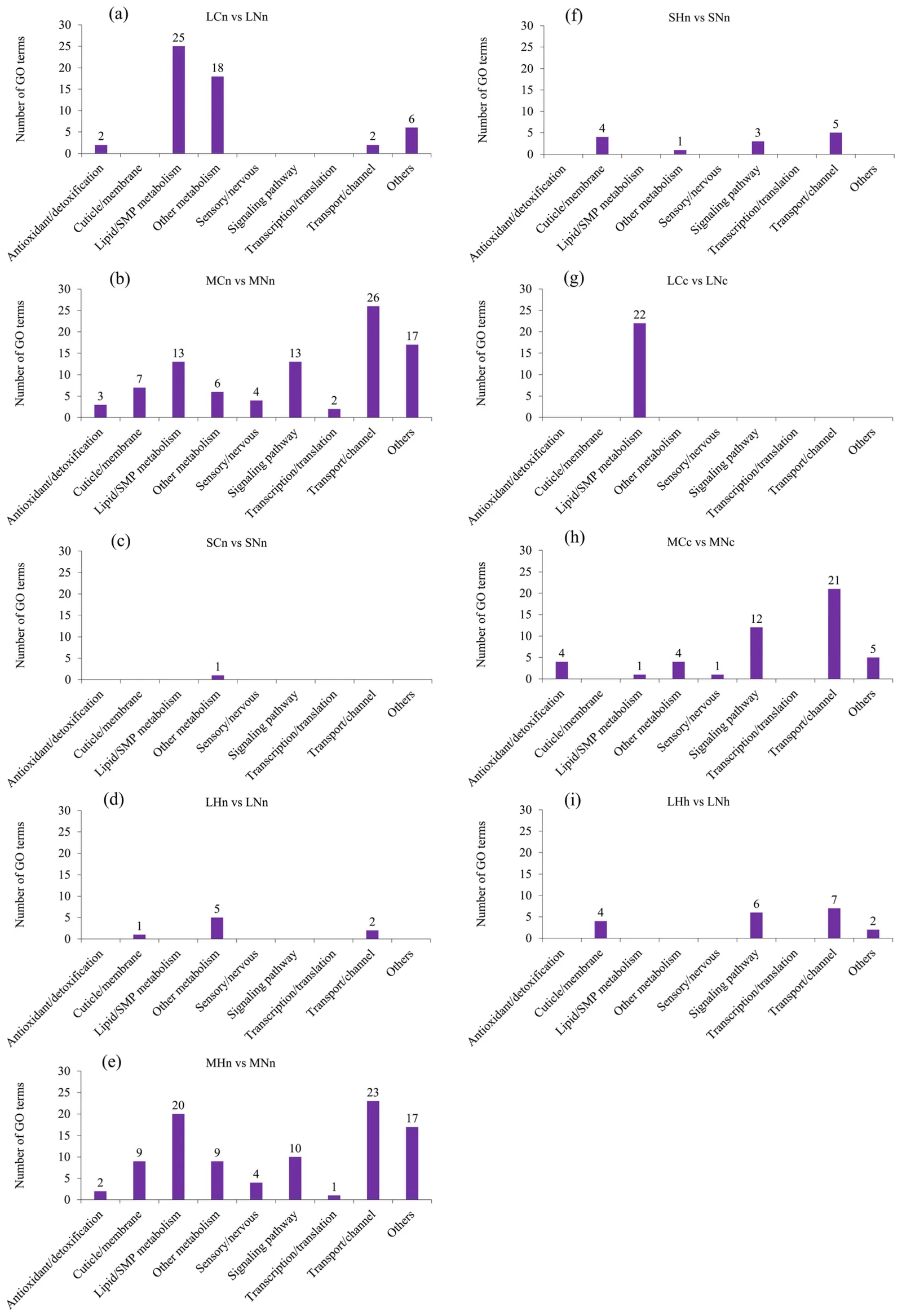
Enriched GO terms for different comparison groups. (**a**–**c**) represent terms of cold-acclimated large, medium and small worker ants (compared with control worker ants with the same body size), respectively; (**d**–**f**) represent terms of heat-acclimated large, medium and small worker ants (compared with control worker ants with the same body size), respectively; (**g**,**h**) represent terms of cold-acclimated and then cold-stressed large and medium worker ants (compared with cold-stressed control worker ants with the same body size), respectively; (**i**) represents terms of heat-acclimated and then heat-stressed large worker ants (compared with heat-stressed control large worker ants).

**Figure 3:**
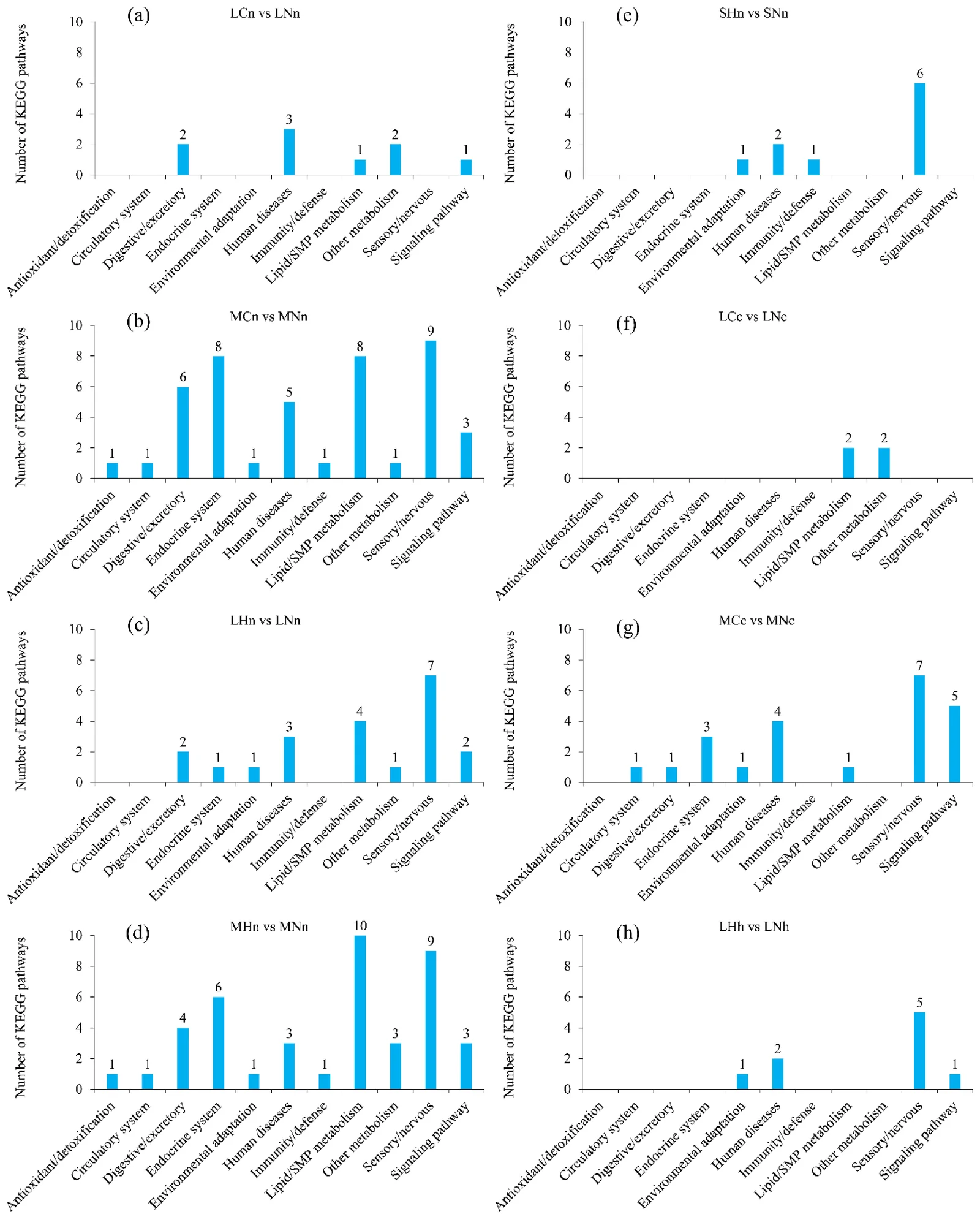
Enriched KEGG pathways for different comparison groups. (**a**,**b**) represent pathways of cold-acclimated large and medium worker ants (compared with control worker ants with the same body size), respectively; (**c**–**e**) represent pathways of heat-acclimated large, medium and small worker ants (compared with control worker ants with the same body size), respectively; (**f**,**g**) represent pathways of cold-acclimated and then cold-stressed large and medium worker ants (compared with cold-stressed control worker ants with the same body size), respectively; (**h**) represents pathways of heat-acclimated and then heat-stressed large worker ants (compared with heat-stressed control large worker ants).

In the group of cold-acclimated large workers vs. control large workers (LCn vs. LNn; “L” refers to large workers, “C” refers to cold-acclimated, “N” refers to control, and “n” refers to no cold stress; i.e., individuals received cold acclimation but were not exposed to extreme cold stress), a total of 586 DEGs were identified (Figure S2a; Table S5), which were enriched in 53 GO terms (Figure 2a; Table S6) and 9 KEGG pathways (Figure 3a; Table S7), including 25 lipid/small molecule protectant (SMP) metabolism-related terms, 18 other metabolism-related terms, as well as 3 human disease-related pathways.

In the group of cold-acclimated medium workers vs. control medium workers (MCn vs. MNn; “M” refers to medium workers), a total of 2643 DEGs were identified (Figure S2b), which were enriched in 91 terms (Figure 2b) and 44 pathways (Figure 3b), including 3 antioxidant/detoxification, 7 cuticle/membrane, 13 lipid/SMP metabolism, 6 other metabolism, 4 sensory/nervous, 13 signaling pathway, and 26 transport/channel-related terms, as well as 6 digestive/excretory, 8 endocrine system, 5 human diseases, 8 lipid/SMP metabolism, 9 sensory/nervous, and 3 signaling pathway-related pathways.

In the group of cold-acclimated small workers vs. control small workers (SCn vs. SNn; “S” refers to small workers), a total of 21 DEGs were identified (Figure S2c), which were enriched in 1 term (Figure 2c) but not in any pathways.

**Figure 4:**
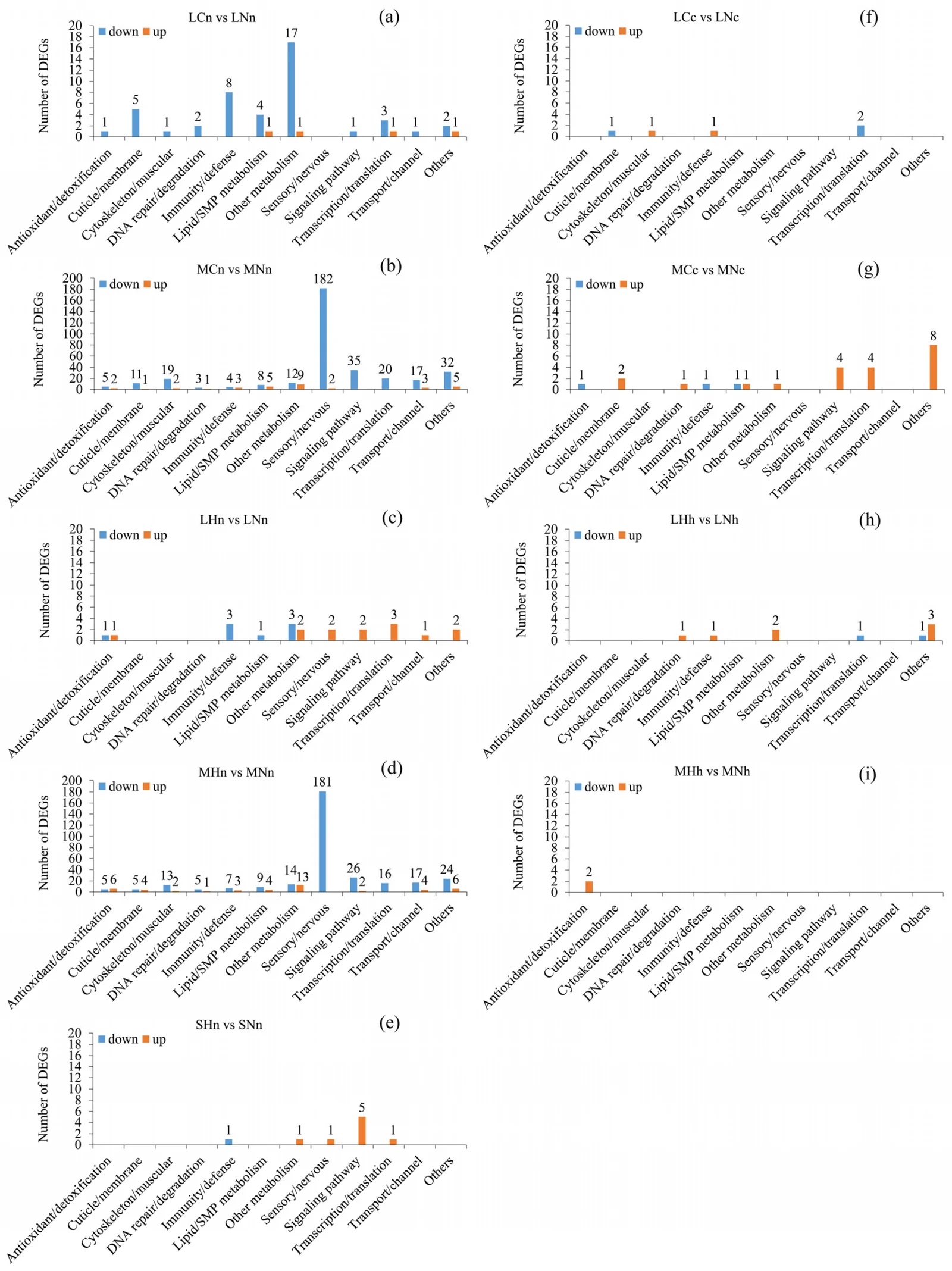
DEGs with highly significant changes (*q* < 0.05, |log2FC| > 3) in different comparison groups. (**a**,**b**) represent DEGs of cold-acclimated large and medium worker ants (compared with control worker ants with the same body size), respectively; (**c**–**e**) represent DEGs of heat-acclimated large, medium and small worker ants (compared with control worker ants with the same body size), respectively; (**f**,**g**) represent DEGs of cold-acclimated and then cold-stressed large and medium worker ants (compared with cold-stressed control worker ants with the same body size), respectively; (**h**,**i**) represent DEGs of heat-acclimated and then heat-stressed large and medium worker ants (compared with heat-stressed control worker ants with the same body size), respectively. Blue columns denote downregulated DEGs, whereas orange columns represent upregulated DEGs.

In the group of cold-acclimated large workers plus cold stress vs. control large workers plus cold stress (LCc vs. LNc; “c” refers to cold stress; i.e., individuals received cold acclimation and then were exposed to extreme cold stress), a total of 102 DEGs were identified (Figure S2d), which were enriched to 22 terms (all related to lipid/SMP metabolism; Figure 2g) and 4 pathways (Figure 3f).

In the group of cold-acclimated medium workers plus cold stress vs. control medium workers plus cold stress (MCc vs. MNc), a total of 643 DEGs were identified (Figure S2e), which were enriched in 48 terms (Figure 2h) and 23 pathways (Figure 3g), including 4 antioxidant/detoxification, 4 other metabolism, 12 signaling pathway, and 21 transport/channel-related terms, as well as 3 endocrine system, 4 human diseases, 7 sensory/nervous, and 5 signaling pathway-related pathways.

In the group of cold-acclimated small workers plus cold stress vs. control small workers plus cold stress (SCc vs. SNc), a total of 7 DEGs were identified (Figure S2f) that were not enriched in any terms or pathways.

### 3.4. Heat Acclimation and Stress-Induced Transcriptional Changes and Functional Enrichment Analysis

In the group of heat-acclimated large workers vs. control large workers (LHn vs. LNn; “H” refers to heat-acclimated; i.e., individuals received heat acclimation but were not exposed to extreme heat stress), a total of 238 DEGs were identified (Figure S3a; Table S5), which were enriched in 8 terms (Figure 2d; Table S6) and 21 pathways (Figure 3c; Table S7), including 5 other metabolism-related terms, as well as 3 human diseases and 7 sensory/nervous-related pathways.

In the group of heat-acclimated medium workers vs. control medium workers (MHn vs. MNn), a total of 2815 DEGs were identified (Figure S3b), which were enriched in 95 terms (Figure 2e) and 42 pathways (Figure 3d), including 9 cuticle/membrane, 20 lipid/SMP metabolism, 9 other metabolism, 4 sensory/nervous, 10 signaling pathway, and 23 transport/channel-related terms, as well as 4 digestive/excretory, 6 endocrine system, 3 human diseases, 10 lipid/SMP metabolism, 3 other metabolism, 9 sensory/nervous, and 3 signaling pathway-related pathways.

In the group of heat-acclimated small workers vs. control small workers (SHn vs. SNn), a total of 212 DEGs were identified (Figure S3c), which were enriched in 13 terms (Figure 2f) and 10 pathways (Figure 3e), including 4 cuticle/membrane-, 3 signaling pathway-, and 5 transport/channel-related terms, as well as 6 sensory/nervous-related pathways.

In the group of heat-acclimated large workers plus heat stress vs. control large workers plus heat stress (LHh vs. LNh; “h” refers to heat stress; i.e., individuals received heat acclimation and then were exposed to extreme heat stress), a total of 186 DEGs were identified (Figure S3d), which were enriched in 19 terms (Figure 2i) and 9 pathways (Figure 3h), including 4 cuticle/membrane-related, 6 signaling pathway-related, 7 transport/channel-related terms, as well as 5 sensory/nervous-related pathways.

In the group of cold-acclimated medium workers plus heat stress vs. control medium workers plus heat stress (MHh vs. MNh), a total of 10 DEGs were identified (Figure S3e) that were not enriched in any terms or pathways.

In the group of cold-acclimated small workers plus heat stress vs. control small workers plus heat stress (SHh vs. SNh), a total of 1 DEG was identified (Figure S3f), which was also not enriched in any terms or pathways.

### 3.5. Clustering and Expression Heatmap Analysis of DEGs with Highly Significant Changes

**Figure 5:**
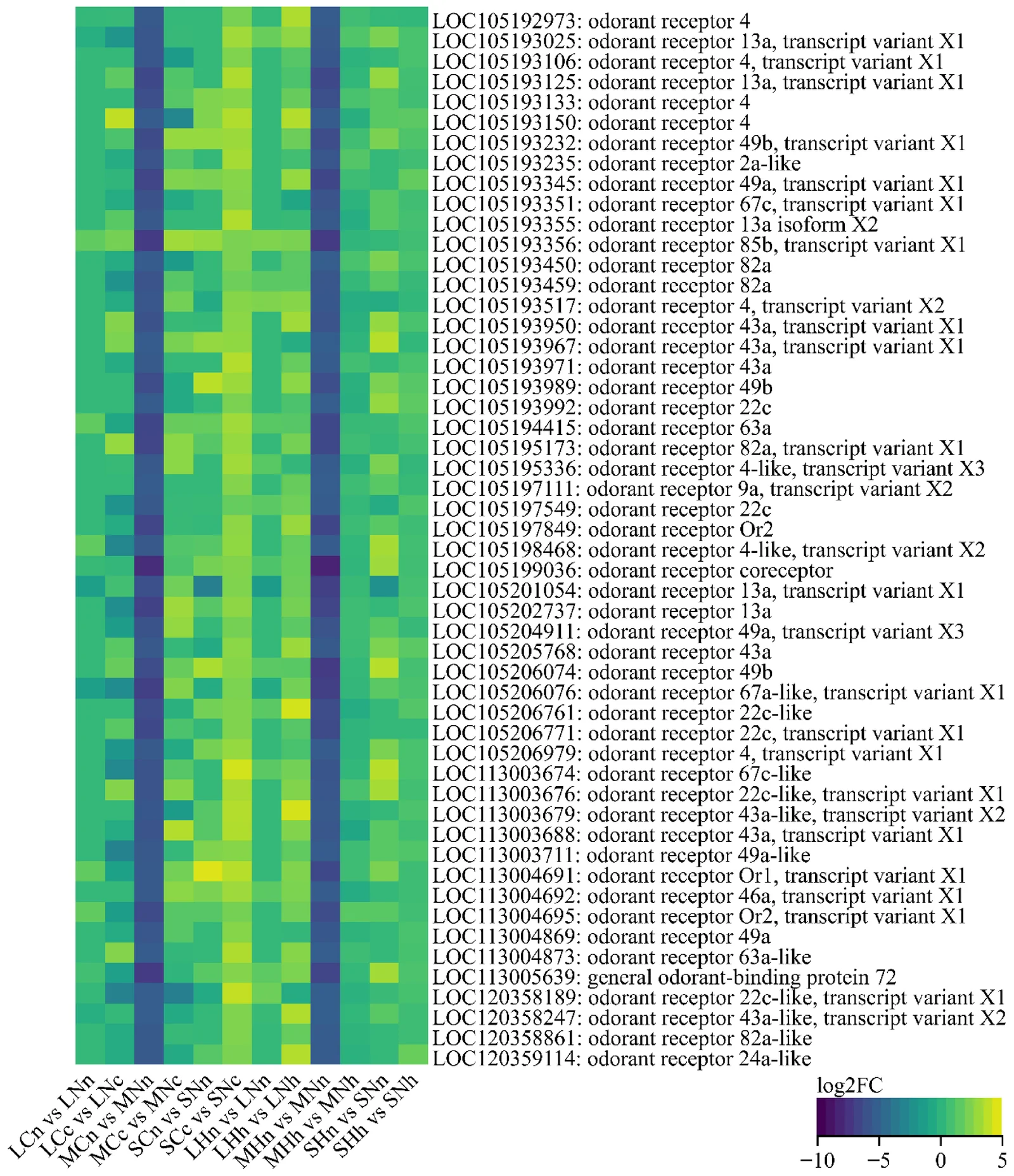
Expression heatmap of highly downregulated odorant receptor-encoding genes (these genes exhibited *q* < 0.05 and log_2_FC < −5.5 in both MCn vs. MNn and MHn vs. MNn comparisons).

DEGs with highly significant changes (*q* < 0.05, |log2FC| > 3) were further identified and analyzed (Figure 4; Table S8). It was shown that a large number of such DEGs were detected in MCn vs. MNn (33 up, 348 down) and MHn vs. MNn (45 up, 322 down), followed by LCn vs. LNn (4 up, 45 down), MCc vs. MNc (21 up, 3 down), and LHn vs. LNn (13 up, 8 down), while other groups showed fewer (<10 for each) of such DEGs. Functional annotation found 177 and 178 highly downregulated (−8.5837 to −3.0140) odorant receptor-encoding genes in MCn vs. MNn and MHn vs. MNn groups, respectively (Table S8), and most (168) of them were shared by the two groups. Among the 168 shared genes, 45 exhibiting log2FC < −5.5 in both comparisons are shown in Figure 5. In addition, a considerable number of metabolism (lipid/SMP metabolism, other metabolism), signaling pathway, transport/channel, cytoskeleton/muscular, and transcription/translation related such highly changed DEGs were found in some comparison groups, including LCn vs. LNn, MCn vs. MNn, and MHn vs. MNn, with the majority of them being downregulated.

### 3.6. Validation of RNA-Seq Sequencing Data

**Figure 6:**
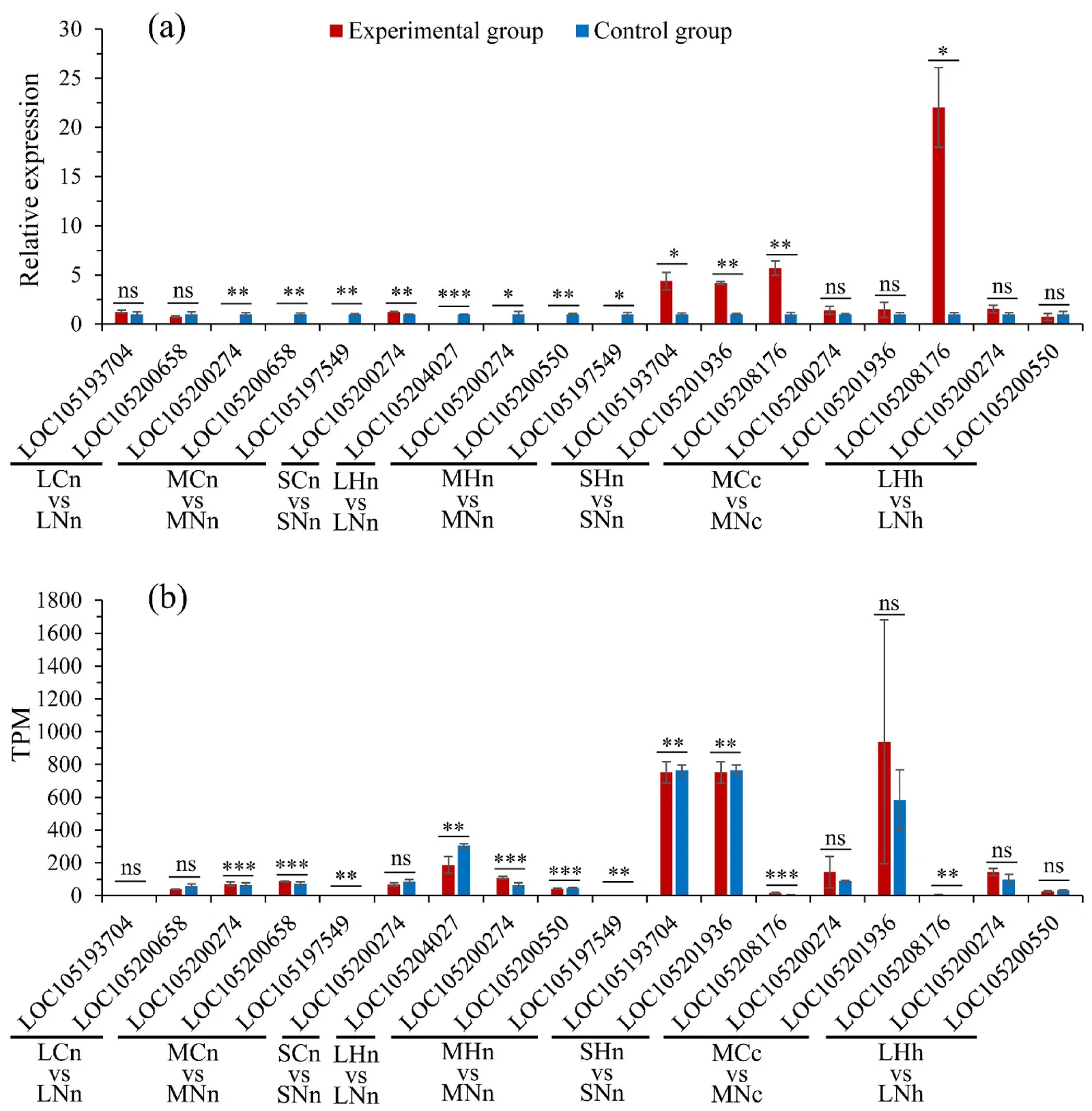
Validation of RNA-seq data using qPCR. (**a**) Expression levels of target genes determined by qPCR. “ns” indicates *p* > 0.05, * indicates *p* < 0.05; ** indicates *p* < 0.01, *** indicates *p* < 0.0001; (**b**) Corresponding results obtained from RNA-seq analysis. “ns” indicates *q* > 0.05, ** indicates *q* < 0.01, *** indicates *q* < 0.0001. TPM: transcripts per million.

A total of 18 genes (12 DEGs and 6 non-DEGs) were selected from the six comparison groups to validate the accuracy of RNA-seq. The results showed that the expression levels of target genes from qPCR (Figure 6a) were similar to the results from the RNA-seq analysis (Figure 6b), suggesting that the RNA-seq data were reliable.

## 4. Discussion

Chen et al. [46] reported that *S. invicta* outperforms *S. richteri* in resistance to high temperature and desiccation, and that thermal tolerance differs between castes, with major workers being more heat-resistant than minor ones. A recent study in *S. invicta* further revealed that cold and heat stress exert significant impacts on the survival of *S*. *invicta* workers, whereby smaller individuals suffer higher mortality rates compared to larger nestmates [12]. In the present study, we show that cold acclimation significantly enhanced the cold tolerance of *S. invicta* workers, while heat acclimation significantly enhanced the heat tolerance of workers. We also observed a correlation between body size and thermal acclimation, whereby acclimated medium-sized workers exhibited significantly higher survival rates than acclimated small workers. However, in large workers, no significant differences were found between acclimated and control groups, with both showing very low mortality under extreme temperatures. This may be because large workers have higher tolerance potential than small and medium workers, and wild-type individuals can tolerate these set extreme temperatures. Body size is frequently positively associated with thermal tolerance in invertebrates [11,41,42,50,51], while neutral and negative relationships have also been observed [11,41]. For example, smaller-sized individuals of *Daphnia magna* display greater heat tolerance as well as higher heat acclimation potential [59]. For ants specifically, larger morphs generally possess enhanced resistance to desiccation and temperature stress [12,46,60,61,62,63]. Larger insects exhibit a lower surface area-to-volume ratio and thus lose water more slowly than smaller individuals [47,64], and they generally possess more fat bodies together with greater reserves of lipids, proteins and glycogen that enable them to sustain elevated energy demands under stress [45,65,66]. In *S. invicta*, workers supplied with sucrose solution exhibited the lowest water loss and mortality under temperature stress [12], presumably because sucrose intake meets elevated energy requirements during stress, generates metabolic water upon decomposition to cut water loss, and facilitates water balance maintenance via modulating hemolymph osmotic regulation [67,68]. In *S. invicta*, division of labor is associated with both worker age and body size: younger, smaller workers primarily engage in brood care, whereas older, larger workers tend to perform foraging [69]. Consequently, larger workers may exhibit superior adaptation to environmental fluctuations and greater thermal tolerance. Nevertheless, further investigations using more extreme low- and high-temperature regimes are required to elucidate the acclimation potential of large workers.

Thermal acclimation is well known to reshape insect thermal tolerance [15,18,19]. Field surveys on insects have demonstrated that ambient thermal stress acts as a selective force facilitating rapid adaptive evolution [70,71]. Highly migratory and dispersive insect species endure greater thermal stress, triggering strong selection and rapid evolutionary change [71,72]. Laboratory research on insect thermal responses lays theoretical groundwork for predicting their adaptation and dispersal trends [73,74]. A laboratory study on *Ischnura elegans* damselflies indicated that pretreatment with cold temperatures mimicking sudden cold events boosted cold tolerance, matching field evidence of enhanced cold resistance in larvae at expansion fronts [75,76]. A thermal pre-acclimation experiment conducted at temperatures ranging from 20 to 30 °C revealed that the heat tolerance of the fall armyworm first-instar larvae and adults rose as acclimation temperature increased [77]. A recent investigation also demonstrated the potential essential contribution of gut microbes to cold tolerance in the fall armyworm [72]. In natural habitats, however, cross-talk between temperature and co-occurring stressors (humidity, pathogens, predators) complicates insect thermal acclimation patterns [74,78,79]. Additional multi-factor field research is thus needed to evaluate the adaptation and invasion expansion of *S. invicta*.

Cumulative molecular research has further revealed that thermal stress remodels genome-wide transcriptional profiles in insects, including ants [12,29,37]. High-throughput sequencing in recent studies has advanced our comprehension of heat stress responses and thermal acclimation processes. For instance, comparative transcriptomic and proteomic analyses of a highly heat-adapted strain versus a conventional strain of the predatory mite *Neoseiulus barkeri* identified 5374 DEGs and 500 differentially expressed proteins, whereby Hsps showed elevated expression as a conserved response, whereas several protective enzymes (SOD, GST, P450, and peroxidase) were down-regulated [80]. For *S. invicta*, prior studies have reported that several-hour cold or heat stress triggers body size- and temperature-specific transcriptional alterations; smaller workers exhibit more DEGs than larger counterparts, suggesting that small workers are likely to incur greater temperature stresses, which is consistent with the observation of size-dependent reductions in worker survival [12,46]. In the present study, both 15-day cold and heat acclimations, as well as subsequent thermal stress, substantially reshape transcriptional profiles of workers in a size-specific manner: medium workers exhibit a higher number of DEGs than large and small workers. Functional enrichment of these DEGs highlighted transport/channel, lipid/SMP metabolism, signaling, and cuticle/membrane-related GO terms, as well as sensory/nervous, lipid/SMP metabolism, and endocrine-system KEGG pathways. Insect cuticles have evolved primarily to prevent transcuticular water loss and pathogen invasion, while also conferring resistance to abiotic stress [81,82]. Temperature modulates cell membrane fluidity, with heat increasing fluidity and cold inducing rigidification, and maintaining optimal membrane fluidity is critical for sustaining normal biomembrane physiological functions [83]. Under heat stress, organisms tend to enhance biosynthesis of saturated fatty acids and sterols to counteract excessive membrane fluidity [29,83]. Unsaturated fatty acids can inhibit crystallization of membrane lipids at low temperatures; consistent with this, cold exposure upregulates FAD9, a key biosynthetic enzyme for unsaturated fatty acids in the tobacco armyworm [84]. Small-molecule cryoprotectants such as trehalose, glycerol, and sugars increase cellular bound water and stabilize enzymes and proteins, thereby improving insect cold tolerance. Studies in ants and tobacco armyworms highlight the vital roles of GPDH and GK in glycerol biosynthesis and cold adaptation [36,37]. Consistently, wheat midges exposed to cold display peak trehalose concentrations and elevated trehalose synthase expression, accompanied by reduced transcripts of trehalose catabolic enzymes [38]. Transporters and ion channels are indispensable for insect adaptation to temperature stress. Thermal perturbation disrupts membrane fluidity and intracellular osmotic equilibrium, inducing transcriptional reprogramming of channel and transporter genes to restore ion gradients and facilitate transport of compatible solutes and metabolic substrates [15,32,85,86].

Prior studies have commonly demonstrated that heat stress triggers large-scale transcriptional alterations in Hsps [29,87]. In the fall armyworm, short-term thermal stress (40 °C for 3 h) significantly induced upregulation of numerous Hsps in both sexes, with 16 Hsps upregulated in females and 26 in males [29], whereas heat selection over four successive generations enhances thermal acclimation and transcriptional responses of Hsps to heat stress in adult males [15]. Cold shock also induces the expression of Hsps in insects, particularly small Hsps (sHsps). For example, Hsp19.5, Hsp20.8 and Hsp21.7 are critical for cold tolerance in the American leafminer [88]. In *Drosophila*, Hsp67 and Hsp23 prevent misfolding and aggregation of damaged proteins, while Hsp67 also mediates recovery from cold shock-induced coma to protect against frostbite [89]. In *S. invicta*, short-term heat stress also induced expression changes in a large number of Hsps, including some sHsps, whereas cold stress affected only a small number of Hsps [12,90]. In the present study, however, after 15 days of temperature acclimation, merely 3 Hsp DEGs (2 upregulated, 1 downregulated) were detected in MCn vs. MNn, and 4 Hsp DEGs (2 upregulated, 2 downregulated) in MHn vs. MNn, all exhibiting modest fold changes (|log2FC| < 2). Furthermore, subsequent cold or heat stress did not strengthen their transcriptional responses. This phenomenon may arise either from sufficient early-stage overexpression of Hsps [12] to sustain later thermal tolerance, or from species-specific adaptive strategies for long-term thermal stresses. Nevertheless, further research is required to test this hypothesis, such as implementing multigenerational assays.

The above enrichment analysis significantly enriched quite a number of sensory/nervous-related terms and pathways (Figure 2 and Figure 3). Further clustering and expression heatmap analysis of DEGs with highly significant changes further identified hundreds of largely downregulated (−8.5837 to −3.0140) odorant receptor-encoding genes in both cold- and heat-acclimated worker ants (Figure 4). Odorant receptor genes encode olfactory receptor proteins that detect volatile chemical signals and mediate chemosensory perception in insects [91,92]. Olfaction serves as the core chemical communication system in ants and other social insects, mediating nestmate discrimination, foraging, and coordination of colony activities, and odorant receptors are vital to maintain normal social behaviors [93,94]. Cold and heat stresses usually suppress foraging and long-distance chemical communication behaviors, which may reduce the physiological demand for olfactory detection and the expression of olfactory-related proteins [95,96,97]. Research on three ant pest species, *Camponotus floridanus*, *Atta sexdens*, and *A. cephalotes*, has uncovered differential expression of olfactory-related genes linked to foraging behavior; olfactory receptor genes are generally upregulated in foraging castes compared with non-foraging ones [98]. Differential expression of odorant-binding protein genes is associated with feeding behavior between male and female mosquitoes, and such variation relies on their metabolic status [99,100]. In *Drosophila melanogaster*, electroantennogram (EAG) and single sensillum recording (SSR) analyses reveal elevated EAG amplitudes upon heat exposure and reduced amplitudes under cold treatment, indicating that environmental temperature modulates olfactory reception [101]. A later study in *D. melanogaster* further demonstrated that thermal treatment at 30 °C for 48 h resulted in the upregulation of 52 and downregulation of 43 genes associated with olfactory reception [102]. In *S. invicta*, previous studies also revealed that both cold and heat stress-induced expression changes in olfactory reception encoding genes [12,90]. Moreover, our study also showed that temperature acclimation and stress also induced a considerable number of strongly changed DEGs that were related to metabolism, signaling, transportation, cytoskeleton, transcription and translation, with the majority of them being downregulated. Low and high temperatures are capable of suppressing metabolism [29,103], and reduced metabolic rates could result in trade-offs between stress resistance processes and basic metabolic functions [28]. Collectively, short-term thermal stress can induce immediate large-scale upregulation of numerous genes, particularly those associated with thermal stress response and tolerance [12,90]. In contrast, long-term thermal acclimation (15 days, as examined in this study) leads to broad suppression of diverse functional genes involved in chemosensory, metabolic, signaling and cytoskeletal pathways. These findings provide novel mechanistic evidence that thermal acclimation coordinately modulates olfactory perception and core metabolic homeostasis in ants, revealing a potential physiological relationship between stress tolerance and olfactory-dependent social behaviors.

In summary, this study demonstrates body-size-dependent thermal acclimation in *S. invicta* workers. Medium and small workers attained improved survival under extreme temperatures following acclimation, while large workers displayed high intrinsic thermal resistance. These phenotypic patterns corresponded to divergent transcriptional reprogramming, with medium workers showing the most extensive expression changes. Thermal acclimation and stress elicited transcriptional changes in pathways associated with lipid/cryoprotectant metabolism, membrane homeostasis, ion transport, antioxidant defense, and sensory signaling. Downregulation of odorant-receptor genes suggests potential olfactory suppression, while heat-shock proteins were only mildly modulated. These findings elucidate a body-size-dependent thermal acclimation mechanism in fire ants and improve forecasts of their invasive range expansion under global warming.

## Data Availability

The transcriptome raw reads have been deposited in the NCBI SRA database; the accession number is PRJNA1496565. Other data generated or analyzed during this study are included in this article and its Supplementary Information Files.

## Supplementary Materials

The following supporting information can be downloaded at: https://www.mdpi.com/article/10.3390/insects17090971/s1, Figure S1: The principal component analysis (PCA) for RNA-seq of biological replicates. Figure S2: Volcanic plots of DEGs of the cold acclimation and stress group. (a–c) represent DEGs of cold-acclimated large, medium and small worker ants (compared with control worker ants with the same body size), respectively; (d–f) represent DEGs of cold-acclimated and then cold-stressed large, medium and small worker ants (compared with cold-stressed control worker ants with the same body size), respectively. Blue dots represent significantly downregulated DEGs, red dots denote significantly upregulated DEGs, and gray dots correspond to genes without significant differential expression. Figure S3: Volcanic plots of DEGs of the heat acclimation and stress group. (a–c) represent DEGs of heat-acclimated large, medium and small worker ants (compared with control worker ants with the same body size), respectively; (d–f) represent DEGs of heat-acclimated and then heat-stressed large, medium and small worker ants (compared with heat-stressed control worker ants with the same body size), respectively. Blue dots represent significantly downregulated DEGs, red dots denote significantly upregulated DEGs, and gray dots correspond to genes without significant differential expression. Table S1: Primers for real-time quantitative PCR. Table S2: Results of two-way ANOVA. Table S3: Summary of the quality of all sample sequencing data. Table S4: Pearson’s correlation coefficient. Table S5: DEGs of different comparison groups. Table S6: GO enrichment analysis results. Table S7: KEGG enrichment analysis results. Table S8: DEGs with highly significant changes (*q* < 0.05, |log2FC| > 3).
